# “Advice to the medical students in my service”: the rediscovery of a golden book by Jean Hamburger, father of nephrology and of medical humanities

**DOI:** 10.1186/1747-5341-8-2

**Published:** 2013-03-15

**Authors:** Piccoli Giorgina Barbara

**Affiliations:** 1 Department of Clinical and Biological Sciences, SS Nephrology, University of Torino, ASOU San Luigi Gonzaga, Orbassano Torino, Italy

**Keywords:** History of nephrology, Medical humanities, Jean Hamburger, Medical education, Jean Hamburger (1909–1992)

## Abstract

Jean Hamburger (1909–1992) is considered the founder of the concept of medical intensive care (*réanimation médicale*) and the first to propose the name Nephrology for the branch of medicine dealing with kidney diseases. One of the first kidney grafts in the world (with short-term success), in 1953, and the first dialysis session in France, in 1955, were performed under his guidance. His achievements as a writer were at least comparable: Hamburger was awarded several important literary prizes, including prix Femina, prix Balzac and the Cino del Duca prize (1979), awarded, among others, to Jorge Luis Borges and Konrad Lorenz.

Here we would like to offer a selected reading of a “golden” book, “Conseils aux étudiants en medicine de mon service” (“Advice to the Medical Students in my Service”), the first book dedicated to patient-physician relationship in Nephrology, written when dialysis and transplantation were becoming clinical options (1963). The themes include: the central role of the patient, who should be known by name, profession, life style, and not by disease; the importance of the setting of the care; the need for truth-telling and for leaving hope; the role of research not only in the progression of science, but also in the daily clinical practice.

## Background

**Figure 1:**
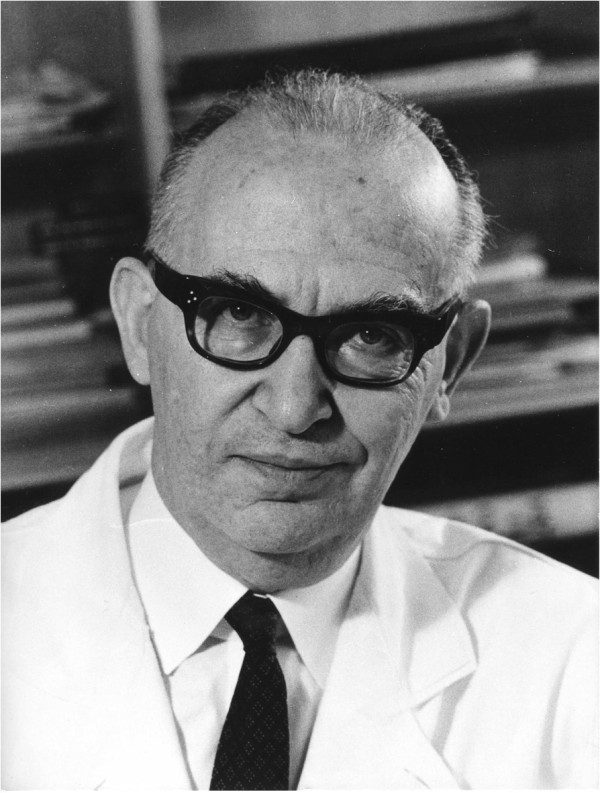
Jean Hamburger; Courtesy of F. Martinez.

Almost every nephrologist of my generation has heard of Jean Hamburger (1909–1992), one of the great masters of our discipline worldwide and the father of French Nephrology [1,2]. For most of us, his name recalls the “*Actualités Néphrologiques de l’Hôpital Neker*”, among the first European meetings focused on a beautiful mix of clinical and research lectures, and the image of an imposing man, almost hidden behind large eyeglasses (Figure 1). How many of us know that he’s considered (at least in France) the inventor of the name Nephrology to identify the branch of medicine dedicated to the care of kidney diseases and of the new concept of “medical intensive care” (*réanimation médicale*), initially closely linked to the development of Nephrology? [1].

*Grand Officier de la Légion d’honneur* (great officer of the *Légion d’honneur)*, member of the highly prestigious *Académie des Sciences *(Academy of Sciences), whose members are called “*les immortels*” the immortals, founder of *la Fondation pour la Recherche Médicale* (Foundation for the Medical Research) and among the first to develop the idea that research is the engine of the medical progress, he was also “*Commandeur de l’Ordre des Arts et des Lettres*” (Commander of the Order of Arts and Literature) [1].

For such individuals, legends and history often merge, sometimes leading to a loss of details or to a dichotomy between different aspects of the personality. For example, probably very few nephrologists know that Jean Hamburger was awarded several important literary prizes, including the *prix Femina Vacaresco* for a literary essay (1972), the *prix Balzac* and the international Cino del Duca prize (both in 1979), awarded among others to Konrad Lorenz and Ignazio Silone, which he received the year after Leopold Sedar Senghor, the great poet from Senegal, and the year before Jorge Luis Borges [1,3].

Hamburger’s great interest in philosophy and the human condition complements, and progressively overtakes, his long list of medical publications. The book list is impressive; while medicine dominates in the first 30 years, from 1935 (*Les Migraines,* Migraines) to the world famous *“Traité de néphrologie”* (Treatise on Nephrology, 1966), in the following almost 30 years, titles such as “*L’Homme et les Hommes”* (Man and Men), *“La Raison et la Passion”* (Reason and Passion), *“La puissance et la fragilité”* (Power and Fragility) or *“Le Livre de l’aventure humaine*” (Book of the Human Adventure) reveal a growing and profound interest in the fragile, complex and fascinating human condition [1,4-9].

At a time when ethics, medical humanities, the patient-physician relationship and holistic approaches are perhaps becoming too common passwords in medicine, the Cartesian humanistic and humane attitude of Jean Hamburger may offer some extraordinary occasions for reflection on the profound implications of a philosophical background in our daily activity as physicians [10,11].

The aim of the present paper is to offer a selected reading on the patient-physician approach, as it appears in the first part of the book that, more than all the others, represents a bridge between the essayist and the nephrologist: “*Conseils aux étudiants en médecine dans mon service”* (éd. Flammarion 1963): “Advice to the Medical Students in my Service” [12].

## The context

The historical context of “*mon service*” can be exemplified by an image of a huge team of clinicians in front of the photographer, among which Jean Hamburger is sitting in the centre, in a discrete albeit dominating position, reported in an historical article on Kidney International [13]. It was in such a context that medicine shifted from art to science. This is depicted in a short, concise and incisive way in the following lines: “Everything started some twenty years ago, when men of my generation became conscious of a disturbing situation: the medicine of our country, which had met with great successes … was in a critical state … our masters, brilliant followers of an age-old tradition, were attached to its cult; they did not appreciate that, in the same period, medicine was undergoing profound modifications. They remained faithful to a sort of literary conception of our discipline, confident in the fact that it represented an art more than a science … it was the time when my master Lemierre preferred to choose his pupils based on a Latin translation …” ([12]; pages 10–13).

## Education for evolution

Education is the means to face this difficult cultural evolution. The need for what is now called “Continuous Medical Education” is clearly envisaged, i.e. the importance of not leaving the updating of knowledge merely to the good will of the individual: “It became evident that post-university teaching”, characterised by being “regular, methodical, almost obligatory, duly organised”, should take the place of “fragmented, insufficient initiatives” undertaken by some well-wishing physicians or groups ([12]; pages 12–13). It was in such a setting that “Advice” was written.

## The patient comes first

The behaviour of a physician in the presence of a patient is the first point dealt with. The (almost revolutionary) novelty is in the beginning: “the first chapter for which you should establish the rules of your actions regards your relationship with the patients”. In keeping with his very inquisitive attitude, he adds: “every physician may define his personal rules on this subject. I’ll share mine with you, not because I find them untouchable, but because they will serve as the basis for reflection and I will demand that you observe them during the period you’ll be in my service.” The Cartesian master then adds: “In any case, I don’t think … it’s enough to trust one’s own instinct and love for others. On the contrary, there is the need for a technique…”.

The first point is that “to understand” patients “one should clearly understand that the patient, lying in his hospital bed, has almost never the same psychological reactions as a normal person”. With this in mind, Hamburger, one of the inventors of modern technical medicine, goes as far as saying that “if we must attempt to create a psychological aid for our patients and to lessen the fear inspired by disease and death, it is not only to give them comfort and aid … but because discomfort and moral pain have the most dangerous effect on the evolution of their organic disease. The psychological medical action has to be considered as important a therapy as dietary or drug prescriptions …”.

## The major rules

The scientific attitude and the humanistic allure are reflected in the following two major and three minor rules. “The first rule is the prohibition against pronouncing in front of a patient some words that experience indicates are possible sources of anxiety”. Interestingly, among the names not to be pronounced, such as death, cancer, coma and autopsy, there is also “lupus”, a disease whose diagnosis does not have such a completely negative meaning today ([12], page 17; [14]).

The second rule “consists in giving to the patients all the desirable explanations of their status, the analyses that are going to be performed, the interventions …”. The reason is clearly stated: “A large part of the fears of our hospitalised patients stems from the ignorance about where they are and what is going on”. There are however some difficulties: “The main one resides in the obligation to tell only the truth, with the risk of losing the confidence of the patients”; thus it is better to announce a painful intervention, explaining its purposes. This rule applies to any medical act, “you should get in the habit of explaining each minimal gesture: this ophthalmoscope … is a simple way to look into your eyes….”. The final goal, by obtaining comments such as: “It was much less painful, Doctor, than you made me think”, is to acquire confidence, identified as fundamental in the medical act. In this regard, Hamburger can be seen as a forerunner of the discovery of the crucial role of compliance, which is deeply rooted in a solid patient-physician trust and relationship ([12], pages 17–19).

This long rule of “patient education” ends with an interesting remark: there is one exception, namely letting patients know that they are affected by an incurable disease. This statement prompts us to reflect on the different concept of “incurable diseases” at present and 50 years ago. Once more, the reason for this act, in apparent contrast with the great role of patient education, is explained by the influence of psychology in the response to diseases: it is not based, Hamburger says, “on religious or metaphysical bases” but simply on the observation that a diagnosis without hope “may prematurely kill, even without the excuse of euthanasia” (ibidem page 23).

## The minor rules

Three “minor rules” are mentioned as possibly neglected but surely important parts of the physician’s behaviour toward patients: calling the patients by their names and not by their diseases. “First of all, it’s important that hospitalised patients do not feel that they are anonymous and deprived of their personality: it’s a great fault to indicate the patient by the bed or room number or by the name of the disease: ‘Here is the slow endocarditis of which I spoke to you this morning’”. And he goes as far as saying that it is also wrong to say “this patient”. Patients should not only be called by surname and first name, but physicians and nurses should know that “Mr. Stephane Dubois is … an art bookbinder, that he loves his job … that he has a charming fiancée, and that he’s a passionate reader of *L’Equipe*”. However (second minor rule), the interest in the person should respect his/her privacy, and he also warns against the use of irony or humour, too often misunderstood by the patients who hide their anxiety behind a “smiling braveness”.

Attention to the family, a valuable resource in healing, is a third rule. The image is clear and sharp. Families are often important, particularly in the case of anxious patients; here the Master criticises the rigid rules “limiting the visiting hours as much as those of convicted prisoners…” and enunciates his freedom of thinking, following the example of anxious patients: “In such cases, one should not hesitate to jostle (*bousculer*) the administrative rules”.

In the last few lines, following the minor rules, Hamburger warns against three great problems: noise, useless long waits and bacterial contamination. With this attention to one great scourge (contamination) and two very important aspects whose effect on the quality of life of hospitalised patients has only recently been acknowledged (noises and long waiting times), the first fundamental chapter of “Advice” is concluded.

It gives way to more technical advice specifically focussed on diagnostic approaches, therapeutic choices and the “personal organization” of study and continuous updating, all vital elements in the development of our discipline, whose reading, albeit interesting, is relevant for the history of the technical development of Nephrology, but is devoid of the timeless message conveyed by the first chapter on the behavioural rules for a physician [12].

## Zouchy, a sort of self-portrait

Jean Hamburger was a fascinating and complex character. There is a sort of self-portrait on the cover page of his book “*Zouchy et quelques autres histoires*” (“Zouchy and other stories”) [15]. This exceptional book, unlike the medical studies and philosophical essays constituting the core of Hamburger’s literary production, gathers a series of novels written during a “forced vacation” in a sanatorium for a young patient “imprisoned” within the same disease. The illustrations are another fascinating surprise: paintings by Eugène Ionesco, the famous playwright, giving a lively description of himself as a man who renounces words to choose paintbrushes instead.

In the following lines, the sick physician and the educated master, a friend of the great talents of his time (the book is dedicated to Henri Flammarion, founder of one of the most important French publishing houses), merge in the most unusual portrait of an ironic and gentle dreamer, a charming model for those among us who are presently searching for examples of how humanities and science can complement each other.

“(…) Twenty-five years ago, a forced vacation had confined me to the mountains for a whole year: I started writing a work of fiction. I created some strange characters that kept me company. My hero tried to distract, during one summer, a sixteen-year-old girl, imprisoned, as I was, by the disease. He told her some extravagant tales. They amused the publisher who presently wishes to publish them. They also seduced a young seventy-year-old painter, who had obstinately continued, until recently, to mainly write theatre-plays: Eugène Ionesco. His sketches, with jubilant virtues, add an unexpected and unhoped-for supplement of dream to my characters”.

## Hamburger’s heritage in Nephrology

The heritage of the masters is always difficult to identify, in particular when it is relatively recent and when it deals with the origins of a discipline, but can we imagine the European Nephrology without Jean Hamburger?

Nephrology would have probably been named kidney medicine, under the strong influence of the American medicine, while the Greek etymology may be seen as a further witness of the strong link between humanities and medicine, of which the whole life of Jean Hamburger is an interesting example [1].

More specifically, the influence of Jean Hamburger was probably too strong to be confined to a single school. His friends and colleagues defined Hamburger as a “Cartesian Master”. This definition applies to being a Master in life (Descartes, Maître de vie, Descartes, Master of life) and to the strict application of logics to any type of problem-solving task. The multifaceted, controversial and rich character of the great master is evident also in the memories reported by the French and French-speaking pioneers of the following generation. One of the very first is Gabriel Richet, his first assistant and another great master of Nephrology. In a recent interview in a project on the Masters of the *Francophone* Nephrology, Richet emphasized Jean Hamburger's role as a teacher and a founder of Nephrology [16].

The French and to a large extent the whole European Nephrology was deeply influenced by the combination of the strict scientific method, grounded in a pathophysiologic logic, in the best French Cartesian tradition, and the careful observation of single cases, based, as the present paper reviews, not only on technical skills, but also on an ethical attitude and respect towards the sick persons [12].

## Competing interest

The author declares that he has no competing interest.

## Author’s contribution

The manuscript has been seen and approved by the author and it is not under consideration for publication elsewhere in a similar form, in any language.
